# Determinants of deranged thyroid function parameters in children admitted for management of diabetic ketoacidosis/diabetic ketosis

**DOI:** 10.1186/s12902-020-00616-2

**Published:** 2020-09-01

**Authors:** Peng Shao, Shujuan Guo, Guimei Li, Daogang Qin, Sen Li, Ying Luan

**Affiliations:** 1 Department of Pediatrics, Shandong Provincial Hospital, Cheeloo College of Medicine, Shandong University, Jinan, 250021 Shandong China; 2 Department of Pediatrics, Liaocheng People’s Hospital, Liaocheng, 252000 Shandong China; 3 Department of Endocrinology, Liaocheng People’s Hospital, Liaocheng, 252000 Shandong China

**Keywords:** Diabetes, Thyroid function, Metabolic changes, Type 1 diabetes, Children

## Abstract

**Background:**

Euthyroid sick syndrome (ESS) frequently arises in children admitted with diabetic ketoacidosis/diabetic ketosis (DKA/DK). This study evaluates the interplay of various metabolic factors with occurrence of deranged thyroid function tests in children suffering from DKA/DK.

**Methods:**

98 DKA and 96 DK pediatric patients were selected from hospital records. Those on thyroxine replacement, with overt hypothyroidism, or with positive anti-thyroperoxidase (TPO) antibody were excluded. Tests for liver function, renal function, lipid profile, serum osmolarity, thyroid function, c-peptide levels, and glycosylated hemoglobin were done on all patients. Children were divided into euthyroid (*n* = 88) and ESS groups (*n* = 106).

**Results:**

The ESS group had a higher level of white blood cell count (WBC), plasma glucose (PG), beta-hydroxybutyric acid (β-HB), triglyceride (TG), anion gap (AG), glycosylated hemoglobin (HbA1c) and a lower level of HCO_3_^−^, prealbumin (PA), and albumin (ALB) compared with the euthyroid group (*P* < 0.05). Free T3 (FT3) levels were significantly correlated to β-HB, HCO_3_^−^, AG, PA, and HbA1c (r = − 0.642, 0.681, − 0.377, 0.581, − 0.309, respectively; *P* < 0.01). Free T4 (FT4) levels were significantly correlated to β-HB, HCO_3_^−^, and ALB levels (r = − 0.489, 0.338, 0.529, respectively; *P* < 0.01). TSH levels were significantly affected by HCO_3_^−^ only (r = − 0.28; P < 0.01). HCO_3_^−^ level was the most important factor deciding euthyroid or ESS on logistic regression analysis (OR = 0.844, *P* = 0.004, 95%CI = 0.751–0.948).

**Conclusions:**

Lower levels of free thyroid hormones and occurrence of ESS were associated with a higher degree of acidosis in children with DKA/DK.

## Background

Type 1 diabetes (T1DM) is a common autoimmune disorder associated with other autoimmune diseases such as coeliac disease and autoimmune hypothyroidism. Although no age group is exempt, children under 18 are most affected.

In children with T1DM due to insulin deficiency, blood ketone levels are higher than normal. Very high ketone levels can trigger a life-threatening condition named diabetic ketoacidosis (DKA), which is manifested by nausea, vomiting, stomach pain, breathing trouble, and loss of consciousness [1, 2]. Infection, stress, inappropriate diet/malnutrition, or medications can all exacerbate DKA, and it has a higher incidence in younger children [3].

Euthyroid sick syndrome (ESS), also known as non-thyroidal illness syndrome, is a transient derangement in thyroid function tests characterized by low T3 levels. ESS is reportedly associated with a higher risk of fatality among critically ill patients admitted with myocardial infarction, sepsis, trauma, and chronic kidney disease [4–7]. In addition, ICU patients with ESS tend to experience more severe symptoms compared to those with normal thyroid function [8]. Thyroid dysfunction occurs more often in diabetic patients relative to the general population while poor glycemic control coincides with a lower level of free T3 (FT3) in serum [9]. ESS in children with T1DM results in poor metabolic control and ketoacidosis [10, 11]. The potential mechanisms include (but are not limited to) deranged regulation of the hypothalamic-pituitary-thyroid axis, inflammatory cytokines effects, and oxidative stress effects [12].

A study reported by Hu et al. revealed a higher level of HbA1c, anion gap, and plasma glucose as well as a lower level of bicarbonate in T1DM children diagnosed with DKA and ESS compared with those without ESS [13]. However, they analyzed the association of just a few of parameters to ESS in DKA. Therefore, the relationship of DKA, ESS, and various metabolic parameters like hyperglycemia, hyperketosis, acidosis, and other acute phase reactants remains to be elucidated.

In this study, we evaluated metabolic parameters such as leukocyte count, blood biochemistry, liver function, kidney function, blood lipids, and C-peptide levels in relation to risk of ESS among children with T1DM admitted for management of DKA, diabetic ketoacidosis (DK), or acute hyperglycemia.

## Methods

### Patient recruitment and exclusion criteria

This was a retrospective case-control study undertaken in the Pediatric Department of Shandong Provincial Hospital affiliated to Shandong University in Shandong, China. We enrolled children with newly diagnosed (or already suffering from) T1DM admitted to the hospital for management of DKA or acute hyperglycemia between January 2014 and August 2019. Ethical approval was obtained from the Ethical Committee of Shandong Provincial Hospital. Study participants and parents of minors provided signed informed consent when they were admitted to the hospital.

T1DM was diagnosed based on the American Diabetes Association criteria and International Society for Pediatric and Adolescent Diabetes (ISPAD) Clinical Practice Consensus Guidelines 2018 [14, 15]. The criteria for diagnosing DKA was blood glucose (BG) > 11 mmol/L, venous pH < 7.3, or bicarbonate < 15 mmol/L. A diagnosis of DK was made if blood glucose was more than 11 mmol/L, urine ketones were positive, but venous pH ≥7.3 or serum bicarbonate level was normal (≥15 mmol/L) [16]. Euthyroidism was defined when levels of thyroid function tests were within the following reference ranges: FT3 3.1–6.8 pmol/L, FT4 12–22 pmol/L, and TSH 0.27–4.2 μIU/mL. ESS was defined when FT3 and/or FT4 levels were decreased and TSH levels were normal or decreased [13, 17, 18].

A total of 237 pediatric patients were screened. All children under the age of 18 years admitted for management of DKA/DK (either newly diagnosed or already on insulin) were included in the trial. Children with overt hypothyroidism, on thyroid medications, or those with other types of diabetes like pancreatic diabetes or juvenile type 2 diabetes, morbid obesity, or on anti-hyperglycemic agents other than insulin were excluded from the trial. Children on steroids or any such medication that could significantly affect thyroid function tests were also excluded from the analyses. Children with other critical organ illnesses as rheumatic heart disease, heart failure, nephrotic syndrome, coeliac disease, or chronic kidney disease as well as those positive for anti-TPO antibody were also excluded.

A total of 194 children qualified for the final analysis: 88 cases in the euthyroid group including 19 cases of DKA and 69 cases of DK, and 106 in the ESS group including 79 cases of DKA and 27 cases of DK.

### Laboratory assays

All samples for laboratory analysis were taken before commencing initial therapy. Serum biochemical analysis including electrolytes, renal function tests, lipid profile, and liver function tests were measured using an automatic biochemistry analyzer (AU5400, Beckman Coulter, Tokyo, Japan). Glycosylated hemoglobin (HbA1c) was measured by high-performance liquid chromatography (HLC-723G7, Tosoh Corporation, Tokyo, Japan). Thyroid function tests were measured using an automated chemi-luminescent immunoassay system (Advia Centaur, Siemens, Munich, Germany). The intra-assay and inter-assay coefficients of variation were < 6% for all parameters. Serum C-peptide (C-p) level was measured by chemi-luminescent immunometric assay (Cobas E170, Roche Diagnostics, Mannheim, Germany). Corrected Na^+^ was derived from this formula: [(Glucose (mmol/L) − 5.6] × 0.36 + Serum Na^+^ [19]].

### Statistical methods

Normally distributed data is represented as mean ± standard deviation (SD). Non parametric and skewed data is represented as median (interquartile range). Chi-squared test was used to compare rates and proportions. Two-tailed student t-tests was used to compare normally distributed data between two groups and Mann-Whitney Rank sum test was used to compare non parametric skewed data between the two groups. Pearson and Spearman correlation tests were applied to evaluate associations of parametric and nonparametric data, respectively. Multiple linear regression analysis was done to detect variables which had an independent effect on thyroid function tests. Logistic regression analysis was done using thyroid function status as a binary variable to determine which factors independently influenced the placement of a subject in a particular group. All analysis was performed on Statistical Package for Social Sciences version 25.0 (SPSS Inc. Chicago, IL, USA). A *p*-value < 0.05 was considered statistically significant.

## Results

### Baseline clinical and laboratory characteristics

The baseline demographic and clinical characteristics of enrolled children are shown in Table 1. There was no statistically significant difference in mean age, gender ratio, and BMI between the euthyroid and ESS groups. White blood cell count (WBC), plasma glucose (PG), beta-hydroxybutyric acid (β-HB), triglyceride (TG), anion gap (AG), and glycosylated hemoglobin (HbA1c) levels were significantly higher in the ESS group than in the euthyroid group, whereas serum HCO_3_^−^, albumin (ALB), and prealbumin (PA) levels were significantly lower in the ESS group than in the euthyroid group (*p* < 0.05).

**Table 1:** Baseline clinical and laboratory characteristics in DKA/DK children

Variables	Thyroid function		Z/T/χ^**2**^	P
	Euthyroidism	ESS		
Gender (male/female)	50/38	59/47	0.03	0.871
Age (years)	7.16 ± 4.24	7.42 ± 4.27	0.416	0.678
BMI (kg/m^2^)	16.93 ± 3.19	15.80 ± 3.07	−1.589	0.116
WBC (× 10^9^/L)	7.25 (6.15 ~ 9.82)	10.72 (7.82 ~ 16.9)	−5.316	< 0.001*
PG (mmol/L)	19.13 (14.97 ~ 27.8)	21.77 (16.89 ~ 29.46)	−3.222	0.001*
BUN (mmol/L)	5.35 (4.48 ~ 6.35)	4.95 (3.93 ~ 6.75)	−0.372	0.71
Cr (umol/L)	31.3 (23.55 ~ 48.63)	34.05 (25.15 ~ 45.64)	−0.765	0.444
β-HB (mmol/L)	2.92 (1.61 ~ 5.22)	6.52 (4.28 ~ 8.46)	−7.375	< 0.001*
K^+^ (mmol/L)	4.25 (3.8 ~ 4.6)	4.2 (3.5 ~ 4.63)	−1.403	0.161
Na^+^ (mmol/L)	133.42 ± 4.72	131.02 ± 5.28	3.309	0.001*
Corrected Na^+^ (mmol/L)	139 (137.2 ~ 141.7)	138.6 (134 ~ 140.6)	−1.405	0.16
Cl^−^ (mmol/L)	99.3 (96 ~ 103)	101 (96 ~ 106.15)	−1.256	0.209
HCO_3_^−^ (mmol/L)	19.15 (16.4 ~ 21.9)	9.55 (6.63 ~ 14.75)	−7.946	< 0.001*
OSM (mOsm/L)	284.39 (279 ~ 289.03)	280.02 (272.05 ~ 286.49)	−0.978	0.328
AG (mmol/L)	19.2 (17.2 ~ 22.15)	22.97 (19.65 ~ 27.53)	−4.857	< 0.001*
ALT (U/L)	14 (12 ~ 18.25)	14 (10 ~ 17.75)	−0.746	0.456
PA (mg/L)	123.93 (99.39 ~ 151)	93.88 (71.97 ~ 123.7)	−5.748	< 0.001*
ALB (g/L)	39.76 ± 4.58	37.58 ± 5.53	2.809	0.006*
GLO (g/L)	23.1 (20.08 ~ 26.78)	22.4 (19.3 ~ 25.95)	−1.594	0.111
TC (mmol/L)	4.4 (3.77 ~ 5.06)	4.14 (3.67 ~ 5.24)	−0.267	0.789
HDL (mmol/L)	1.15 (0.96 ~ 1.44)	1.13 (0.94 ~ 1.36)	−0.769	0.442
LDL (mmol/L)	2.51 (2.06 ~ 2.99)	2.54 (1.94 ~ 3.11)	−0.594	0.553
TG (mmol/L)	0.97 (0.69 ~ 1.31)	1.34 (0.91 ~ 2.19)	−3.724	< 0.001*
HbA1c (%)	11.42 ± 2.49	12.62 ± 2.15	−3.593	< 0.001*
C-p (ng/ml)	0.19 (0.09 ~ 0.46)	0.19 (0 ~ 0.34)	−1.294	0.196

*Abbreviations*: *β-HB* beta-hydroxybutyric acid, *ALB* albumin, *GLO* globulin, *HbA1c* glycosylated hemoglobin, *HDL* high density lipoprotein, *LDL* low density lipoprotein, *OSM* osmolarity, *AG* anion gap, *PA* prealbumin, *PG* plasma glucose, *TC* total cholesterol, *TG* triglyceride, *WBC* white blood cell count, *BMI* body mass index, *BUN* serum urea nitrogen, *Cr* creatinine, *ALT* alanine aminotransferase, *C-p* C-peptide

*P < 0.05 ESS vs. euthyroidism

### Correlating and independent influencing factors of FT3

**Fig. 1:**
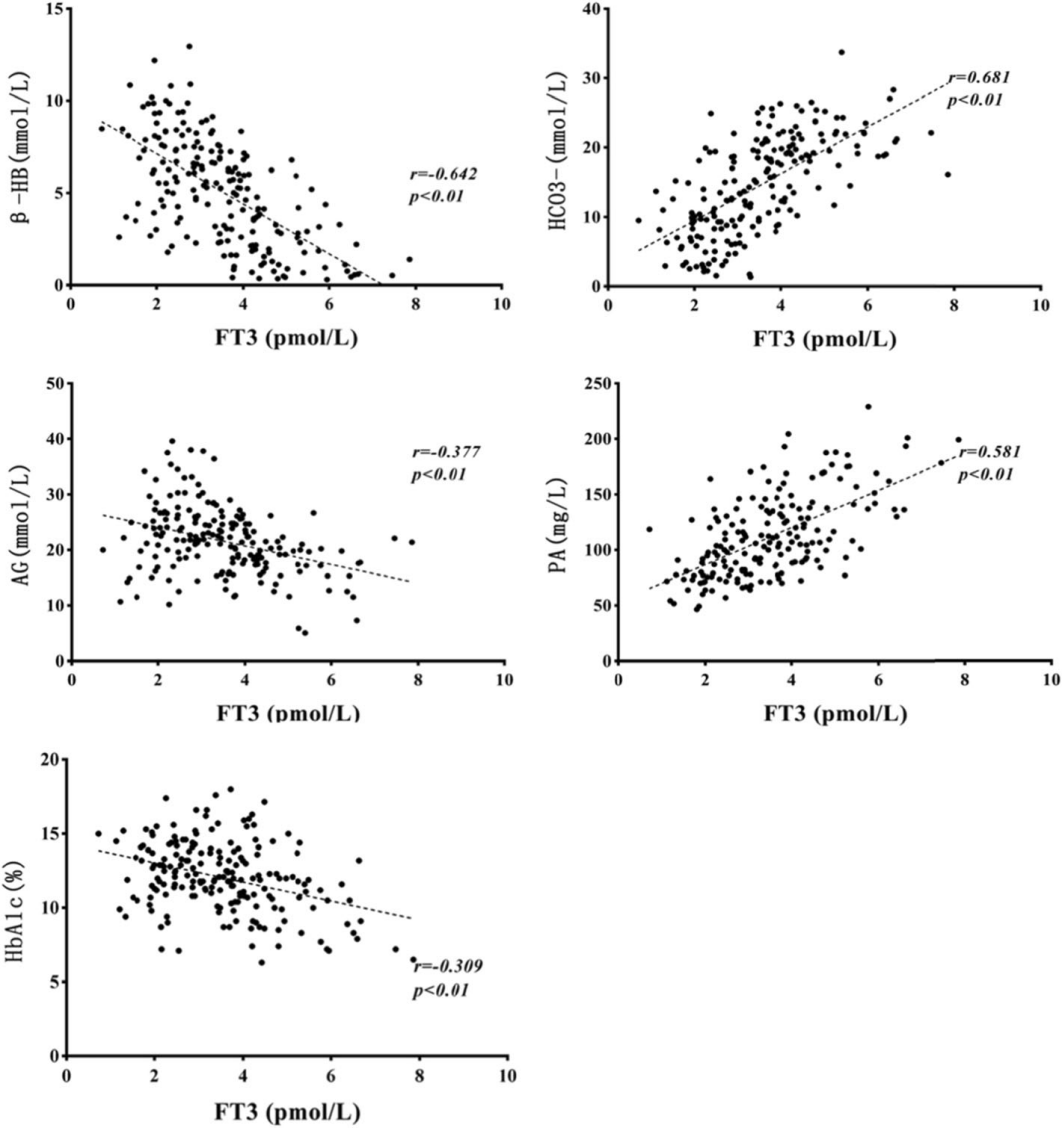
Correlations between FT3 and β-HB, HCO_3_^−^, AG, PA, HbA1c. Abbreviations: β-HB, beta-hydroxybutyric acid; AG, anion gap; PA, prealbumin aminotransferase; HbA1c, glycosylated hemoglobin

FT3 levels positively correlated with levels of serum corrected Na^+^, HCO_3_^−^, PA, K^+^, ALB, GLO, and HDL but negatively correlated with WBC, PG, β-HB, AG, TG, and HbA1c. There was no significant correlation between levels of FT3 and BUN, Cr, Cl^−^, OSM, ALT, TC, LDL, and C-p (*P* > 0.05, data not shown). Variables showing significant correlation were subjected to multivariate linear regression analysis, which showed that β-HB, HCO_3_^−^, AG, PA, and HbA1c independently affected FT3 levels (Table 2). The correlations between these independent variables and FT3 are shown in the scatter plot on Fig. 1 (r = − 0.642, 0.681, − 0.377, 0.581, − 0.309, respectively; *P* < 0.01).

**Table 2:** Correlating factors of FT3 in DKA/DK children

Variables	B	SD	Beta	T	P
WBC	−0.016	0.011	−0.092	− 1.417	0.159
PG	−0.001	0.01	−0.007	−0.122	0.903
β-HB	−0.151	0.048	−0.331	−3.116	0.002*
Corrected Na^+^	−0.006	0.017	−0.02	−0.351	0.726
K^+^	−0.013	0.102	−0.008	− 0.128	0.898
HCO_3_^−^	0.067	0.019	0.342	3.603	< 0.001*
AG	0.042	0.02	0.197	2.161	0.033*
PA	0.011	0.003	0.3	3.961	< 0.001*
ALB	0.02	0.02	0.076	1.04	0.3
GLO	−0.013	0.016	−0.049	− 0.815	0.417
HDL	0.113	0.261	0.028	0.434	0.665
TG	−0.069	0.067	−0.064	−1.031	0.305
HbA1c	−0.07	0.034	−0.121	−2.082	0.039*

*P < 0.05

### Correlating and independent influencing factors of FT4

**Fig. 2:**
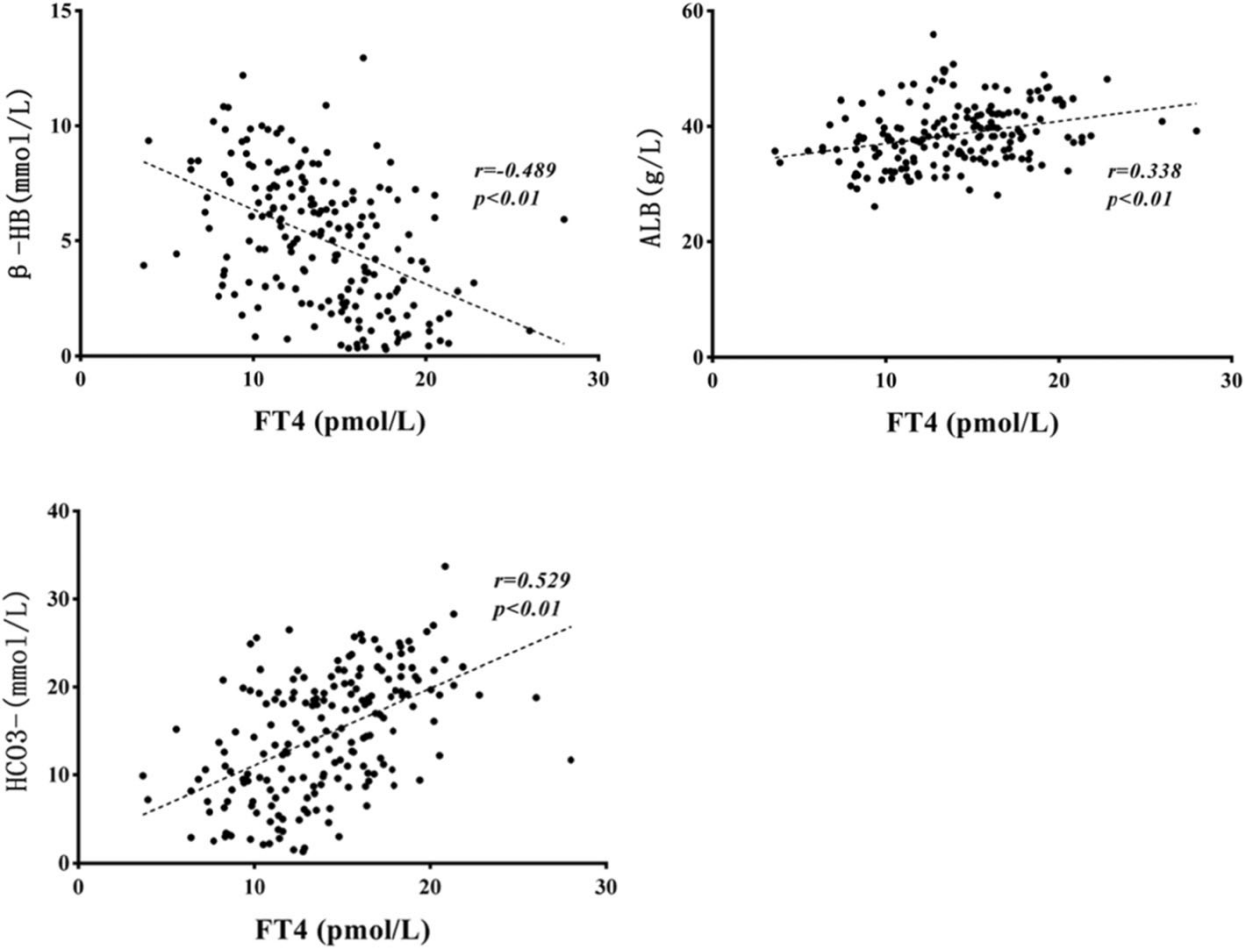
The correlations between FT4 and β-HB, HCO_3_^−^, ALB. Abbreviation: ALB, albumin

FT4 levels positively correlated with serum levels of corrected Na^+^, HCO_3_^−^, PA, ALB, GLO, and HDL but negatively correlated with WBC, PG, β-HB, AG, Cl^−^, TG, and HbA1c. There was no significant correlation between FT4 levels and BUN, Cr, K^+^, OSM, ALT, TC, LDL, and C-p (*P* > 0.05, data not shown). Variables showing significant correlation were subjected to multivariate linear regression analysis, which showed that β-HB, HCO3^−^, and ALB significantly affected FT4 levels (Table 3). The correlations between these variables and FT4 levels are shown in the scatter plot on Fig. 2 (r = − 0.489, 0.338, 0.529, respectively; *P* < 0.01).

**Table 3:** Correlating factors of FT4 in DKA/DK children

Variables	B	SD	Beta	T	P
WBC	−0.009	0.045	−0.017	− 0.199	0.843
PG	−0.003	0.039	0.007	0.086	0.931
β-HB	−0.434	0.196	−0.303	−2.216	0.028*
Corrected Na^+^	0.095	0.077	0.103	1.237	0.218
HCO_3_^−^	0.156	0.084	0.253	1.849	0.047*
AG	0.07	0.082	0.103	0.848	0.398
PA	0.013	0.011	0.119	1.219	0.225
TG	0.135	0.27	0.04	0.501	0.617
HbA1c	0.036	0.136	0.02	0.268	0.789
Cl^**−**^	−0.07	0.039	− 0.158	−1.782	0.077
ALB	0.164	0.078	0.193	2.093	0.038*
GLO	−0.026	0.064	−0.032	− 0.413	0.681
HDL	1.338	1.053	0.107	1.271	0.206

*P < 0.05

### Correlating and independent influencing factors of TSH

**Fig. 3:**
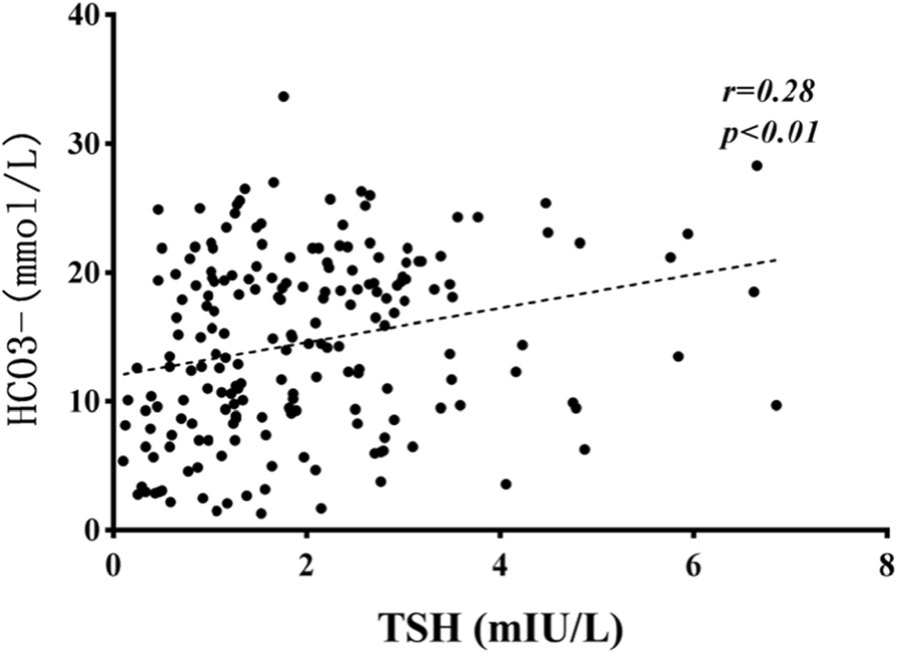
The correlations between TSH and HCO_3_^−^

TSH showed positive correlation with levels of corrected Na ^+^, HCO_3_^−^, OSM, TC, and LDL but negative correlation with WBC, β-HB, and BUN. There was no significant correlation between TSH and levels of PG, K^+^, Cl^−^, Cr, ALT, AG, PA, TG, HbA1c, ALB, GLO, HDL, and C-p (*P* > 0.05), data not shown. Variables which showed significant correlation were subjected to multivariate linear regression analysis, which revealed that only serum HCO3- levels had an independent and significant effect on TSH levels (Table 4). The correlations between serum HCO3^−^ and TSH levels are shown in the scatter plot on Fig. 3 (r = − 0.28, *P* < 0.01).

**Table 4:** Correlating factors of TSH in DKA/DK children

Variables	B	SD	Beta	T	P
WBC	−0.009	0.018	−0.05	− 0.495	0.621
β-HB	0.037	0.059	0.08	0.632	0.529
corrected Na^+^	0.016	0.028	0.052	0.563	0.575
HCO_3_^−^	0.071	0.025	0.362	2.834	0.005*
BUN	−0.025	0.052	−0.042	− 0.493	0.623
OSM	0.005	0.005	0.079	0.876	0.383
TC	0.134	0.262	0.101	0.511	0.61
LDL	0.1	0.35	0.058	0.284	0.777

*P<0.05

### Logistic regression of adjudicated thyroid function group with metabolic covariates

Logistic regression model analysis used the thyroid function subgroup (euthyroid or ESS group) as a non-parametric dependent variable and significantly correlated variables in Table 1 as parametric independent variables. As shown in Table 5, serum HCO_3_^−^ levels was the only factor independently and significantly associated with thyroid function group adjudication (OR = 0.845, *P* = 0.004, 95%CI = 0.752–0.949).

**Table 5:** Logistic regression on the predictors of deranged thyroid function in DKA/DK patients

Variables	B	***p***-value	Wald	Adjusted p-value	OR	95% C.I. for OR	
						Inferior	Superior
WBC	0.127	0.062	4.227	0.050	1.136	1.006	1.283
PG	0	0.028	0	0.996	1	0.946	1.057
β-HB	0.14	0.145	0.937	0.333	1.15	0.866	1.558
HCO3^−^	−0.168	0.059	8.072	0.004*	0.845	0.752	0.949
AG	−0.02	0.066	0.097	0.756	0.98	0.862	1.114
PA	−0.005	0.008	0.436	0.509	0.995	0.979	1.011
TG	0.227	0.267	0.723	0.395	1.255	0.743	2.119
HbA1c	0.179	0.115	2.401	0.121	1.196	0.954	1.499
ALB	−0.071	0.057	1.591	0.207	0.931	0.833	1.04

*P < 0.05

## Discussion

The present work focused on the interplay of metabolic disorders and deranged free thyroid hormone levels in DKA/DK pediatric patients with ESS, which few studies have previously elucidated. The results indicated a poorer glycemic control in these patients as evidenced by higher plasma glucose and glycosylated hemoglobin levels. They also showed a higher degree of acidosis, indicated by higher levels of anion gap and β-HB, as well as a lower level of bicarbonate. Moreover, serum albumin and pre-albumin were reduced in ESS pediatric patients.

Abnormal serum thyroid hormone levels have been well described in patients admitted with acute critical illness [18, 20–22]. The exact underlying causes have not yet been clarified although many hypothesis have been proposed [12, 18, 23, 24]. In previous research, FT3 and FT4 levels were positively correlated with bicarbonate levels and negatively correlated with HbA1c and AG levels [13]. In the present study, we evaluated the role of more parameters including acute phase reactants and metabolic parameters in the specific situation. Linear regression analysis confirmed that β-HB and HCO_3_^−^ levels had an independent influence on both FT4 and FT3 levels while serum pre-albumin and albumin levels related to FT3 alone and FT4 alone, respectively. In addition, HCO_3_^−^ was demonstrated as the only significantly independent influencing factor on TSH.

Previous studies have noticed the relationship between acidosis and free thyroid hormones levels [23, 25]. Indeed, lower T4 and T3 and higher reverse T3 (rT3) serum concentrations were found in patients with metabolic acidosis [25]. Rashidi et al. reported that the lower the pH in DKA patients, the lower the FT3 levels [26]. pH may be the most important factor directly or indirectly affecting iodo-thyronine metabolism and regulation in vitro and in vivo [25]. Our results were consistent with these studies as we also found metabolic acidosis (reflected by serum bicarbonate levels) being the most important factor correlated with parameters of thyroid function tests (i.e. FT3, FT4, and TSH). However, we still could not determine from our data whether ESS is a direct effect of pH or a more general effect of severity of DKA illness.

Many studies have suggested that blood β-HB can be used to rapidly and accurately diagnose DK/DKA in hyperglycemic patients [27, 28]. Measurement of blood ketones has been recommended in national guidelines in UK for assessment of response to therapy and in tailoring insulin infusion rates [29]. However, few studies have detected the relationship between β-HB and thyroid function. Boado et al. found that β-HB may induce a moderate depression of pituitary and plasma TSH in rats [30]. Our study showed a strong negative correlation between levels of β-HB and free thyroid hormone concentrations which may also be the consequence of TSH decline.

As for the underlying reasons for the low serum albumin or pre-albumin in DK/DKA patients with ESS, malnutrition might not be a key factor as there was no significant difference in BMI between ESS and euthyroidism groups in this study. Albumin synthesis in hepatocytes depends on sufficient insulin secretion [31]. It has been demonstrated that insulin deficiency reduces liver albumin production while insulin infusion in diabetic patients enhances live albumin synthesis [32]. In the case of children with longstanding diabetes, the worse compliance and the lower insulin infusion dosing are more likely occurring with ESS. Therefore, in the present study, the more absolute lack of insulin could partly account for the lower level of serum albumin and pre-albumin in T1DM patients with DKA/DK. Moreover, albumin deficiency occurs when DKA/DK pediatric patients are critical ill, and reduced albumin in ESS might indicate a more serious condition.

In the present study, children in the ESS group had higher WBC levels, which were negatively correlated with levels of FT3, FT4, and TSH. Elevated WBC has also been shown to be significantly correlated with severity of DKA and DK in other studies [33, 34]. This phenomenon is most likely a leukemoid-like reaction instead of a systemic inflammatory response, as no evidence for fever or bacteria or virus infection was present. Also, in both adult and pediatric DKA patients, the response to milieu interne changes including deranged hormones, cytokines, and mediators actuates white blood cell count elevation [35].

We also found lower serum sodium in ESS patients, which when corrected by using blood glucose concentration showed no significant difference between the ESS and euthyroidism groups. So, this could be pseudohyponatremia due to the water being osmotically drawn into the vascular space in hyperglycemia. Although low serum sodium concentration has been shown to be indicative of poor outcome or cerebral edema in DKA, the present study did not reveal its relationship with ESS.

Our study had several drawbacks as it was a retrospective study with a small number of participants. We did not measure rT3 levels which are usually elevated in ESS patients. Moreover, we did not correlate ESS with recovery times and hospital stay duration as well as other parameters associated with DKA such as venous thrombosis and cerebral edema.

In addition, as detection limitations, we did not apply equilibrium dialysis or ultrafiltration methods to measure free thyroid hormones in the serum of patients which might underestimate the concentration of FT3 or FT4. This inaccuracy could lead to overdiagnosis of ESS and misclassification, particularly in marginal cases. Although the setting of euthyroidism control partly compensated for this weakness, the impact of this methodology on the outcome might be inevitable.

## Conclusions

Lower levels of free thyroid hormones were found to be associated with higher degree of acidosis and hence with the severity of DKA/DK.

## Data Availability

The datasets are available from the corresponding author on reasonable request.

## Abbreviations

β-HB: beta-hydroxybutyric acid
ALB: Albumin
DK: Diabetic ketosis
DKA: Diabetic ketoacidosis
ESS: Euthyroid sick syndrome
GLO: Globulin
HbA1c: glycosylated hemoglobin
HDL: High density lipoprotein
LDL: Low density lipoprotein
OSM: Osmolarity
PA: Prealbumin
PG: Plasma glucose
TC: Total cholesterol
TG: Triglyceride
WBC: White blood cell count
